# The validity and responsiveness of three quality of life measures in the assessment of psoriasis patients: results of a phase II study

**DOI:** 10.1186/1477-7525-4-71

**Published:** 2006-09-27

**Authors:** Richard Shikiar, Mary Kaye Willian, Martin M Okun, Christine S Thompson, Dennis A Revicki

**Affiliations:** 1 Center for Health Outcomes Research, United BioSource Corporation, Bethesda, MD, USA; 2 Global Health Economics and Outcomes Research, Abbott Laboratories, Abbott Park, IL, USA; 3 Abbott Immunology, Abbott Laboratories, Abbott Park, IL, USA

## Abstract

**Background:**

Patient-reported outcome (PROs) measures are being used more frequently in investigational studies of treatments for moderate to severe plaque psoriasis. The objective of this study was to examine the relationships among the Dermatology Life Quality Index (DLQI), the Short Form 36 (SF-36), and the EuroQOL 5D (EQ-5D) and to assess their validity, responsiveness, and estimates of minimum important differences.

**Methods:**

A Phase II, randomized, double-blind, parallel group, placebo-controlled, multi-center clinical trial assessed the clinical efficacy and safety of two doses of subcutaneously administered adalimumab vs. placebo for 12 weeks in the treatment of 147 patients with moderate to severe plaque psoriasis. This study provided the opportunity to evaluate the validity and responsiveness to change in clinical status of PROs instruments. Patients completed the DLQI, SF-36, and EQ-5D questionnaires at baseline and at 12 weeks. Blinded investigators assessed the Psoriasis Area and Severity Index (PASI) scores and the Physician's Global Assessment (PGA) scores of enrolled patients. The responsiveness of the measures to changes in the clinical endpoints from baseline to Week 12 was assessed. Estimates of minimum important differences (MID) were derived. All analyses were performed with blinded data; findings and conclusions were not biased based on treatment condition.

**Results:**

The dermatology-specific DLQI was highly correlated to clinical endpoints at baseline and at Week 12, and was the most responsive PRO to changes in endpoints. Compared with the SF-36, the EQ-5D index score and VAS scores were generally more highly correlated with clinical endpoints, but displayed about the same degree of responsiveness. The most responsive SF-36 scales were the Bodily Pain and Social Functioning scales. Estimates of the MID for the DLQI ranged from 2.3–5.7 and for the SF-36 Physical Component Summary (PCS) score ranged from 2.5–3.9.

**Conclusion:**

This study provides support for the continued use of the DLQI and SF-36 PCS in the assessment of treatments for psoriasis. On the basis of the results from this trial, the EQ-5D should be considered as a general PRO measure in future clinical trials of patients with moderate to severe plaque psoriasis.

## Background

Moderate to severe plaque psoriasis has been demonstrated to have substantial impact on function limitations and psychosocial factors of patients with the disease [1-5]. Moreover, successful treatment of moderate to severe psoriasis – as assessed by improved physical functioning and reduction of signs and symptoms – has been shown to have a positive impact on social and psychological aspects of psoriasis [6-11].

Given the functional and psychosocial impact of the disease, studies of moderate to severe psoriasis patients often include both physician-assessed clinical endpoints and dermatology-specific patient-reported outcomes (PROs) to obtain a holistic view of the disease and treatment effects in patients [12]. Such practices are bolstered by the assertion of the Medical Advisory Board of the National Psoriasis Foundation (NPF) that, even more so than physical signs, such as the percentage of body surface area (BSA) affected by psoriasis, the severity of psoriasis is "first and foremost a quality-of-life (QOL) issue" [13]. The same values for percentage BSA involvement can result in very different degrees of impact for different patients, depending on the location of psoriatic plaques, the pain associated with the lesions and plaques, the extent of bleeding associated with the psoriatic lesions, and resulting functional limitations. The NPF Advisory Board suggests an alternative basis for defining mild, moderate, or severe psoriasis, predicated on QOL impacts of the disease. Similarly, the guidelines recently promulgated by the British Association of Dermatologists [14] for the use of biologics in psoriasis indicate that eligible patients must have a Psoriasis Area and Severity Index (PASI) score of at least 10 and a score on the Dermatology Life Quality Index (DLQI) [15] – a dermatology-specific validated PRO measure – of greater than 10.

A Phase II clinical trial of two dosages of adalimumab and placebo in the treatment of moderate to severe psoriasis provided an opportunity to further explore the psychometric characteristics – including responsiveness and minimum important differences – of the three PROs used in the trial: the DLQI; the general health-related QOL measure MOS Short Form 36 (SF-36) Health Survey [16]; and the general health status measure EuroQOL 5D (EQ-5D) [17,18]. Establishing the reliability, validity, and responsiveness of PRO measures is necessary for their use in support of labeling claims, according to an FDA draft guide to industry [19]. Reliability refers to the accuracy of a measure, while validity refers to the extent the measure actually is measuring what it purports to measure. Responsiveness is a component of validity and represents the PRO's capability to detect changes related to changes in the clinical status of patients or other relevant outcomes measures. Minimum important difference (MID) is related to responsiveness and provides guidance to those reviewing clinical trial results as to whether the statistically significant group differences or changes are clinically meaningful and important. Jaeschke and colleagues [20] define a minimal clinically important difference (MCID) (we use MID instead of MCID to avoid confusion) as "the smallest difference in score ... which patients perceive as beneficial and which would mandate, in the absence of troublesome side-effects and excessive cost, a change in the patient's management." Estimation of the MID – using several different approaches – is also emphasized in the FDA guidance and is consistent with recently published recommendations of health outcomes researchers [21,22].

## Methods

### Overview

The objectives of the Phase II, randomized, double-blind, parallel group, placebo-controlled, multi-center clinical trial were to assess the clinical efficacy and safety of subcutaneously administered adalimumab vs. placebo using two dosage regimens for 12 weeks in the treatment of 147 patients with moderate to severe plaque psoriasis. The study included a screening period, a blinded 12-week treatment period, and a 30-day follow-up visit for patients not completing 12 weeks of active treatment or not entering an extension study. Time between screening and baseline visits was not to exceed 28 days. The trial achieved the objectives of the study in terms of safety and clinical efficacy endpoints [23].

### Patients and inclusion criteria

Patients with a diagnosis of moderate to severe plaque psoriasis and an affected BSA of ≥ 5% for at least 1 year were eligible for the study. In addition to other inclusion criteria (e.g., age ≥ 18 years, willingness to give informed consent), patients had to be able to self-inject medication or have a designee or nurse who could inject the randomized assignment. Patients signed informed consent forms, and the study complied with FDA Good Clinical Practices, Health Protection Branch guidelines, and all other applicable ethical, legal, and regulatory requirements [23].

### Clinical measures

For purposes of the analyses reported here, there were two primary clinical outcomes:

### Psoriasis Area and Severity Index

Frequently used as an endpoint in psoriasis clinical trials [24], the PASI [25] was the primary efficacy outcome in this trial. PASI is a composite index indicating the severity of the three main signs of psoriatic plaques (i.e., erythema, scaling, and thickness) and is weighted by the amount of coverage of these plaques in the four main body areas (head, trunk, upper extremities, and lower extremities). PASI scores range from 0–72, with higher scores indicating greater disease severity. PASI was assessed at screening and baseline, at Weeks 1, 2, 4, 8, and 12/Early Termination, and at the final follow-up visit.

### Physician's Global Assessment

The PGA is a seven-point scale used to measure the severity of disease at the time of the physician's evaluation. The seven disease categories are:

• *Severe*: very marked plaque elevation, scaling, and/or erythema

• *Moderate to Severe*: marked plaque elevation, scaling, and/or erythema

• *Moderate*: moderate plaque elevation, scaling, and/or erythema

• *Mild to moderate*: intermediate between moderate and mild

• *Mild*: slight plaque elevation, scaling, and/or erythema

• *Almost Clear*: intermediate between mild and clear

• *Clear*: no signs of psoriasis (post-inflammatory hypopigmentation or hyperpigmentation could be present).

The PGA scale is scored from 1 (Clear) to 7 (Severe). The PGA was assessed by the investigator at screening, baseline, and Weeks 1, 2, 4, 8, 12/Early Termination, and the follow-up visit. Each study site was to make every attempt to have the same investigator perform these assessments throughout the study for each patient.

### Patient-Reported Outcome measures

Three PROs measures were used in the study and are the subject of the analyses reported here. All PROs measures were assessed at baseline and at Week 12 (or early termination, if applicable).

### Dermatology Life Quality Index

The DLQI was developed as a simple, compact, and practical questionnaire for use in dermatology clinical settings to assess limitations related to the impact of skin disease [15]. The instrument contains 10 items dealing with skin (e.g., Item 1: "Over the last week, how itchy sore, painful, or stinging has your skin been?"). The DLQI score ranges from 0–30, with "30" corresponding to the worst quality of life, and "0" corresponding to the best score. The DLQI has well-established properties of reliability and validity in the dermatology setting [15,26-28].

### Short Form 36 health survey

The SF-36 is a 36-item general health status instrument often used in clinical trials and health services research [16]. It consists of eight domains: Physical Function, Role Limitations-Physical, Vitality, General Health Perceptions, Bodily Pain, Social Function, Role Limitations – Emotional, and Mental Health. Two overall summary scores can be obtained – a Physical Component Summary (PCS) score and a Mental Component Summary (MCS) score [29]. The PCS and MCS scores range from 0–100, with higher scores indicating better health. The SF-36 has been used in a wide variety of studies involving psoriasis, including descriptive studies [30] and clinical research studies [6,7], and has demonstrated good reliability and validity. Internal consistency for most SF-36 domains is greater than 0.70. The SF-36 has been shown to discriminate between known groups in a variety of diseases, is reproducible, and is responsive to longitudinal clinical changes.

### EuroQOL 5D

The EQ-5D [17,18] is a six-item, preference-based instrument designed to measure general health status. The EQ-5D has two sections: The first consists of five items to assess degree of physical functioning (mobility, self-care, usual activities, pain/discomfort, and anxiety/depression). Items are rated on a three-point scale ranging from "No Problem" to "Extreme Problem" or "Unable to Do." Each pattern of scores for the five items is linked to an index score that has a value ranging from 0–1, indicating the health utility of that person's health status. The specific linkage can differ from country to country, reflecting differences in cultures to the item responses. The second section is the sixth item on the EQ-5D, which is a visual analog scale with endpoints of "100" or "Best Imaginable Health," and "0" or "Worst Imaginable Health." It offers a simple method for the respondents to indicate how good or bad their health statuses are "today." The score is taken directly from the patients' responses.

### Statistical methods

Validity of the PRO measures was assessed in several ways. First, an assessment was made of the concurrent validity of scales and subscales (i.e., the extent to which PRO measures are correlated with one another). As a disease-specific PRO measure, the DLQI was expected to correlate moderately to extremely well with general PRO measures. Another important aspect of validity in this study was to assess the extent to which the PRO measures correlated with the clinical endpoints – PASI and PGA – both at baseline and at Week 12.

Responsiveness of PRO measures was assessed via two approaches. First, changes in these measures from baseline to Week 12 were correlated with changes in the PASI or PGA over the 12-week course of treatment within the trial. Concurrent improvement in both clinical measures and PRO measures was expected to result in positive correlations. The second approach to assessing responsiveness involved categorizing patients into responder groups based on the changes in their PASI scores from baseline to Week 12. This was done in two ways. First, a responder was defined as a patient with >75% improvement in PASI (consistent with the definition of success with the primary efficacy variable), and a non-responder was defined as a patient with a PASI improvement <50% (consistent with the definition of failure for a secondary efficacy variable). Tests of mean differences in improvement on the PRO measures were completed between the two groups. Secondly, in support of the estimation of the MID, discussed below, patients were further categorized by degree of PASI response, and assessed differences among these four groups: PASI improvement <25%; PASI improvement 25–49%; PASI improvement 50–74%; and PASI improvement ≥ 75%. Analyses of variance tests were performed among these four groups for changes in PRO measures.

In accordance with the FDA draft guidance [19] and consistent with recent recommendations from PRO researchers [21,22], five methods were used to estimate MIDs of the PROs. The PRO change score corresponding to PASI 25-PASI 49 was the first estimate of MID, called MID-1. This was based on the assumption that patients would perceive a PASI improvement of 25% as beneficial. The trial did not provide data to test this assumption (e.g., there was no rating by patients of their overall improvements). A second estimate, MID-2, was based on the PRO change score corresponding to a PASI improvement between 50–74%. The PASI 50 is seen as clinically relevant, and, as such, this degree of improvement served as a secondary efficacy endpoint in this trial. A third method for estimating MID relied on the association of changes in the PRO measure with changes in the PGA. A non-responder was defined as a patient with a PGA change score of either "0" (no change) or "1" (slight increase in severity of disease) from baseline to Week 12. A minimal responder was defined as a patient whose PGA improved by either 1 or 2 points from baseline to Week 12. The third estimate of MID, MID-3, was the difference in the PRO score between non-responders and minimal responders.

In addition, two distributional methods were used to support the anchor-based MID estimates for the PROs [21,22]. Based on evidence by Wyrwich and associates [31,32], the standard error of measurement (SEM) can be used to approximate the MID. The SEM, which describes the error associated with the measure, was estimated by the standard deviation of the measure multiplied by the square root of 1 minus its reliability coefficient. Finally, there has been discussion [33] concerning a number of studies demonstrating that one-half of the standard deviation of a measure represents the upper limit of the MID [22]. In estimating the SEM for the SF-36 and the EQ-5D, reliability estimates from the literature were used. The SEM for the DLQI incorporated the reliability estimated from the trial data, which was consistent with what has been found in the literature for this instrument [27].

Finally, it is important to note that all analyses were performed with blinded data (i.e., the statuses of patients with respect to their assigned treatment groups were not known).

## Results

### Patient demographics and clinical characteristics

A total of 147 patients enrolled and received at least one dose of study medication at 18 sites in the United States and Canada. Blinded data were available for the PROs for 147 patients at baseline and 140 patients at Week 12. Since the focus of these analyses were on the psychometric properties of the PROs rather than with efficacy, observed cases were employed rather than last observation carried forward or other methods for treating missing observations at the end of trial. The mean age of the patients enrolled in the trial was 44.2 years, two-thirds were male, and the preponderance were white (Table 1).

**Table 1:** Baseline demographic characteristics

**Characteristic**	**(N = 147)**
**Age**	
Mean (SD)	44.2 (12.7)
**Gender**	
Female n (%)	48 (32.7%)
Male n (%)	99 (67.3%)
**Race**	
White n (%)	133 (90.5%)
Black n (%)	4 (2.7%)
Asian n (%)	5 (3.4%)
Other n (%)	5 (3.4%)

### Clinical endpoints

The results for the PASI and the PGA at baseline and Week 12, as well as the change from baseline to Week 12, are displayed in Table 2. The mean PASI at baseline was 15.7, which decreased by 8.9 points (improvement) to 6.8 by Week 12. The mean PGA at baseline was 5.5 (i.e., midway between "Moderate" and "Moderate to Severe"), and decreased (improved) by 2.1 points to 3.4 by Week 12 (i.e., between "Mild" and "Mild to Moderate"). In evaluating the improvement in the two clinical endpoints, it is important to keep in mind that these analyses included pooled placebo and active treatment groups.

**Table 2:** Mean (Standard Deviation) of PASI and PGA at baseline and week 12, and change from baseline to week 12

**Measure**	**Baseline**	**Week 12**	**Change^2^**
**PASI**	15.69 (7.34)	6.84 (7.77)	-8.87 (8.41)
**PGA^1^**	5.48 (0.81)	3.36 (1.74)	-2.14 (1.87)

^1 ^Scored such that 1 = "Clear" to 7 = "Severe."

^2^Change scores are computed only for the 140 patients with scores at baseline and Week 12; sample size at baseline = 147.

### Patient-Reported Outcome measures

The results for the DLQI, SF-36, and EQ-5D at baseline and Week 12, and the change from baseline are shown in Table 3. Based on blinded data, mean PRO measures improved during the course of the trial (a decrease in DLQI scores indicates an improvement; an increase in the SF-36 and EQ-5D indicates improvement). The greatest improvement in a DLQI item occurred for the first item, assessing how "itchy, sore, painful, or stinging" the person's skin felt (data not shown). Similarly, as shown in Table 3, the greatest improvement among the SF-36 scales was for Bodily Pain. The largest improvement among the five EQ-5D dimensions occurred for the Pain/Discomfort dimension (data not shown). Given these findings, it appears that improvement in pain and discomfort is the most pronounced among all PRO measures assessed.

**Table 3:** Mean (Standard Deviation) of PROs at baseline and week 12, and change from baseline to week 12

**Measure**	**Baseline**	**Week 12**	**Change^1^**
**DLQI Total Score**	12.71 (7.18)	5.28 (6.49)	-7.45 (7.78)
**SF-36**			
Physical Functioning	79.26 (24.95)	84.43 (22.62)	5.63 (22.25)
Role-Physical	72.45 (37.56)	82.50 (34.67)	9.64 (40.30)
Bodily Pain	59.58 (25.37)	75.87 (24.89)	16.59 (26.90)
General Health	66.7 (20.6)	72.69 (20.79)	6.10 (16.25)
Vitality	53.89 (22.81)	61.04 (23.18)	7.60 (20.88)
Social Functioning	74.49 (27.56)	85.98 (23.63)	11.16 (27.80)
Role-Emotional	76.03 (35.39)	85.48 (30.77)	8.39 (37.02)
Mental Health	69.39 (19.30)	77.43 (17.73)	8.14 (18.57)
Physical Summary Score (PCS)	47.93 (10.23)	51.24 (9.51)	3.47 (9.30)
Mental Summary Score (MCS)	47.30 (11.23)	51.36 (10.08)	3.94 (11.04)
**EQ-5D**			
Index Score	0.66 (0.28)	0.82 (0.23)	0.16 (0.29)
VAS Overall Health	72.25 (20.67)	81.22 (17.26)	9.35 (20.71)

^1^Change scores are computed only for patients with scores at baseline and Week 12; this number varied between 138 and 140, depending on the specific measure, as compared with the 147 patients at baseline.

The reliability of the DLQI, as assessed by coefficient alpha, was 0.89 at baseline and 0.92 at Week 12, indicating that this is a highly reliable measure, and in line with previous findings [27,28].

### Relationships among Patient-Reported Outcome measures

Table 4 displays the correlations among PRO measures at baseline and at Week 12, as well as the correlations among changes in these measures from baseline to Week 12. There were a few trends evident form this data. First, all measures were statistically significantly inter-correlated. Second, with respect to the relationship between the DLQI and the SF-36, the DLQI correlated the greatest with the Bodily Pain and Social Functioning domains, both at baseline and at Week 12, and, for changes in these scores over the course of the trial. Third, the DLQI correlated highly with the EQ-5D index score, and these correlations were consistently higher than the correlations with the EQ-5D visual analog scale (VAS) scores. Fourth, the EQ-5D index score tended to correlate greatest with the Bodily Pain domain of the SF-36. Finally, the scores tended to be more highly correlated at the end of the trial than at baseline, consistent with previous findings [28].

**Table 4:** Correlations^1 ^among PROs at baseline and week 12, and change from baseline to week 12

**Measure**	**Baseline**			**Week 12**			**Change**		
	**DLQI Total**	**EQ-5D Index**	**EQ-5D VAS**	**DLQI Total**	**EQ-5D Index**	**EQ-5D VAS**	**DLQI Total**	**EQ-5D Index**	**EQ-5D VAS**
**DLQI Total Score**	1.00	-0.51	-0.35	1.00	-0.71	-0.58	1.00	-0.53	-0.46
**SF-36**									
Physical Functioning	-0.44	0.58	0.35	-0.41	0.61	0.49	-0.29	0.47	0.32
Role-Physical	-0.45	0.64	0.38	-0.57	0.67	0.50	-0.47	0.51	0.45
Bodily Pain	-0.55	0.73	0.45	-0.61	0.76	0.56	-0.66	0.64	0.53
General Health	-0.24**	0.39	0.47	-0.38	0.59	0.69	-0.33	0.46	0.46
Vitality	-0.31	0.43	0.48	-0.43	0.62	0.60	-0.46	0.37	0.48
Social Functioning	-0.69	0.52	0.46	-0.68	0.74	0.60	-0.68	0.50	0.56
Role-Emotional	-0.41	0.45	0.42	-0.56	0.67	0.53	-0.50	0.41	0.48
Mental Health	-0.44	0.46	0.50	-0.56	0.66	0.67	-0.52	0.49	0.56
Physical Summary Score (PCS)	-0.41	0.64	0.36	-0.46	0.65	0.52	-0.41	0.56	0.39
Mental Summary Score (MCS)	-0.45	0.39	0.49	-0.58	0.66	0.63	-0.59	0.42	0.57
**EQ-5D**									
Index Score	-0.51	1.00	0.39	-0.71	1.00	0.63	-0.53	1.00	0.39
VAS-General Health	-0.35	0.39	1.00	-0.58	0.63	1.00	-0.46	0.39	1.00

^1^All correlations were significant at p < 0.001, unless otherwise noted. *p ≤ 0.05, **p ≤ 0.01, ns = non-significant.

### Correlations with clinical endpoints

Table 5 displays correlations of PRO measures with the two clinical assessments – PASI score and PGA – at baseline (first two columns of data) and at Week 12 (second two columns). In addition to almost uniformly greater correlations at Week 12 vs. at baseline – consistent with previous findings [28] – one can also note that both the DLQI and EQ-5D index score tended to be more highly correlated with the two clinical endpoints than any of the SF-36 domains. The SF-36 scales with the strongest association with clinical endpoints are Social Functioning and Bodily Pain.

**Table 5:** Correlations^1 ^between PROs and clinical endpoints at baseline and week 12, and change from baseline to week 12

**Measure**	**Baseline**		**Week 12**		**Change**	
	**PASI**	**PGA**	**PASI**	**PGA**	**PASI**	**PGA**
**DLQI Total Score**	0.31	0.29	0.67	0.65	0.69	0.71
**SF-36**						
Physical Functioning	-0.32	-0.14 ns	-0.28	-0.25**	-0.38	-0.14 ns
Role-Physical	-0.22**	-0.14 ns	-0.41	-0.37	-0.42	-0.31
Bodily Pain	-0.36	-0.19*	-0.47	-0.42	-0.60	-0.44
General Health	-0.08 ns	0.05 ns	-0.34	-0.33	-0.34	-0.24**
Vitality	-0.15 ns	-0.06 ns	-0.37	-0.37	-0.38	-0.31
Social Functioning	-0.23**	-0.21**	-0.46	-0.38	-0.44	-0.43
Role-Emotional	-0.16 ns	-0.06 ns	-0.37	-0.29	-0.39	-0.36
Mental Health	-0.17*	-0.09 ns	-0.46	-0.38	-0.43	-0.36
Physical Summary	-0.28	-0.13 ns	-0.35	-0.33	-0.45	-0.25**
Mental Summary	-0.12 ns	-0.08 ns	-0.44	-0.36	-0.40	-0.42
**EQ-5D**						
Index Score	-0.40	-0.31	-0.60	-0.51	-0.57	-0.44
VAS-General Health	-0.24**	-0.09 ns	-0.52	-0.43	-0.43	-0.38
						
**PASI**	1.00	0.59	1.00	0.83	1.00	0.75

^1^All correlations were significant at p < 0.001, unless otherwise noted. *p ≤ 0.05, ** p < 0.01, ns = non-significant.

### Responsiveness of the Patient-Reported Outcome measures

An important attribute for a PRO measure is responsiveness to change in the clinical status of a patient (i.e., as a patient's disease improves, the PRO measures also improve). The last two columns of Table 5 display the correlations between changes in PRO measures used in the trial and changes in PASI scores and the PGA from baseline to Week 12. These data demonstrate that the DLQI is the most responsive of the PRO measures. The correlations between changes over the course of the trial in the DLQI total score and changes in the PASI score (r = 0.69, p < 0.001) and PGA (r = 0.71, p < 0.001) approach the correlation between changes in the two clinical measures themselves (r = 0.75, p < 0.001). In addition, the DLQI is the only one of the PRO measures to demonstrate equal responsiveness to PGA and PASI scores. The correlation between changes in the EQ-5D index score and the two clinical assessments was r = -0.57 (p < 0.001) for changes in the PASI to r = -0.44 for changes in the PGA (p < 0.001). Similarly, the correlations between changes in all but one of the SF-36 scores and changes in the PGA were smaller than correlations between changes in the SF-36 and the PASI.

A second way to assess responsiveness was to contrast patients who were defined as clinical responders with those characterized as non-responders. Given that the primary endpoint in the trial was defined as the percentage of patients achieving a PASI 75 response (i.e., ≥ 75% improvement in PASI from. baseline) by Week 12, a responder was defined as a patient with a PASI75 response. A non-responder was a patient with <PASI 50, since some of the secondary endpoints in the trial used this cut-off. The results of these analyses are displayed in Table 6. DLQI total scores for responders improved by 12.17 points, while scores of non-responders improved by 1.77 points. This difference was statistically significant (t = 9.0; p < 0.0001). All the PRO measures except for the SF-36 Physical Functioning domain were responsive, as defined by a statistically significant difference between responders and non-responders. The DLQI was the most responsive of the PRO measures, as evidenced by the size of the t-statistic and the effect size. The responsiveness of the EQ-5D index and VAS scores were generally the same as several of the SF-36 domain scores.

**Table 6:** Change in PRO measures among responder^1 ^groups

**Change in Measure**	**Mean Change Score for Responders (n = 66)**	**Mean Change Score for Non-Responders (n = 53)**	**Difference**	**t-value**	**P Value**	**Effect Size**
**DLQI Total Score**	-12.17 (6.78)	-1.77 (5.52)	-10.39	9.0	<.0001	0.40
**SF-36**						
Physical Functioning	9.12 (23.50)	3.52 (20.19)	5.59	1.4	0.1724	0.01
Role-Physical	20.08 (35.69)	-5.19 (44.76)	25.26	3.4	0.0008	0.08
Bodily Pain	26.47 (27.40)	4.21 (22.74)	22.26	4.7	<.0001	0.15
General Health	8.87 (15.62)	1.47 (17.77)	7.39	2.4	0.0178	0.04
Vitality	13.01 (22.58)	1.13 (18.20)	11.87	3.1	0.0024	0.07
Social Functioning	21.59 (28.13)	-2.59 (25.04)	24.19	4.9	<.0001	0.16
Role-Emotional	19.70 (32.54)	-9.62 (36.95)	29.31	4.6	<.0001	0.14
Mental Health	14.55 (17.77)	-0.38 (18.15)	14.92	4.5	<.0001	0.14
Physical Summary Score (PCS)	5.35 (9.67)	1.47 (9.08)	3.88	2.2	0.0287	0.03
Mental Summary Score (MCS)	8.03 (10.59)	-2.03 (10.42)	10.06	5.1	<.0001	0.17
**EQ-5D**						
Index Score	0.25 (0.30)	0.04 (0.26)	0.22	4.2	<.0001	0.12
VAS General Health	15.69 (18.96)	1.92 (23.24)	13.77	3.5	0.0006	0.09

^1^Responder is defined as PASI improvement ≥ 75%; non-responder is defined as PASI improvement <50%.

While the estimates of responsiveness displayed in the last two columns of Table 5 take into account the full range of PASI change scores and their relationship to PRO change scores, the responsiveness analysis in Table 6 places patients in two categories – responders and non-responders. Table 7 defines *four *categories of responders: responders, defined as those with PASI improvements ≥ 75%; "partial responders," those with PASI improvement 50–74%, inclusively; "near responders," those with PASI improvement 25–49%, inclusively; and non-responders, with <PASI25. One-way analyses of variance were performed among these groups for each of the PRO measures. As can be seen by the size of the f-statistics, the DLQI was the most responsive of the PRO measures. In fact, only the DLQI was able to demonstrate statistically significant differences between responders and partial responders based on post-hoc significance tests among the four responder groups. These results for the DLQI total score with respect to differences among responder groups were similar to those reported previously in the literature, except that the improvement in DLQI total scores displayed in Table 7 was larger for each of the responder groups than for the equivalent responder groups described by Shikiar and colleagues in a study of efalizumab [28]. As was the case for the data displayed in Table 6, the responsiveness of the EQ-5D index and VAS scores were generally the same as for most of the SF-36 scores. Finally, both the SF-36 MCS and PCS scores were responsive, but the MCS was substantially more responsive, indicating that the impact of the disease was both physical and mental, with the latter perhaps being more prominent for this study population.

**Table 7:** PRO change scores corresponding to levels of PASI improvement

**Change in Measure**	**PASI Improvement <25% (n = 31)**	**PASI Improvement 25–49% (n = 22)**	**PASI Improvement 50–74% (n = 21)**	**PASI Improvement ≥75% (n = 66)**	**Overall F Value**	**p Values^1^**
**DLQI Total Score**	-0.16 (5.41)	-4.05 (4.95)	-6.95 (5.71)	-12.17 (6.78)	30.4***	2**,3***,5***,6*
**SF-36**						
Physical Functioning	2.15 (17.67)	5.45 (23.60)	0.02 (22.36)	9.12 (23.50)	1.2	
Role-Physical	-11.29 (39.18)	3.41 (51.35)	14.29 (31.20)	20.08 (35.69)	4.9**	3**
Bodily Pain	-1.03 (21.42)	11.59 (22.96)	16.76 (22.74)	26.47 (27.40)	9.0***	3***
General Health	-0.61 (17.33)	4.41 (18.37)	9.19 (11.29)	8.87 (15.62)	2.8*	
Vitality	-1.45 (16.29)	4.77 (20.44)	6.90 (17.43)	13.01 (22.58)	3.8*	3*
Social Functioning	-3.23 (23.71)	-1.70 (27.36)	13.10 (17.44)	21.59 (28.13)	8.7***	3***, 5**
Role-Emotional	-11.11 (36.44)	-7.58 (38.40)	17.46 (34.35)	19.70 (32.54)	7.6***	2*, 3**, 5*
Mental Health	-0.77 (18.08)	0.18 (18.66)	9.52 (13.53)	14.55 (17.77)	7.2***	3**,5*
Physical Summary	-0.31 (7.18)	3.91 (10.88)	2.57 (7.78)	5.35 (9.67)	2.7*	
Mental Summary	-2.19 (9.86)	-1.82 (11.38)	6.05 (6.90)	8.03 (10.59)	9.9***	2*, 3***, 5**
**EQ-5D**						
Index Score	-0.01 (0.26)	0.10 (0.24)	0.20 (0.21)	0.25 (0.30)	7.1***	3***
VAS General Health	0.58 (24.31)	3.82 (22.07)	8.43 (11.24)	15.69 (18.96)	4.8**	3**
						
Mean PASI Improvement^2^	0.94 (4.07)	-6.24 (2.99)	-8.94 (3.47)	-14.33 (7.65)		

^1^Pairwise comparisons between means were performed using Scheffe's test adjusting for multiple comparisons. 1 = improvement <25% vs. improvement 25–49%, 2 = improvement <25% vs. improvement 50–74%, 3 = improvement <25% vs. improvement ≥ 75%, 4 = improvement 25–49% vs. improvement 50–74%, 5 = improve 25–49% vs. improvement ≥ 75%, and 6 = improvement 50–74% vs. improvement ≥ 75%. *p < 0.05, **p < 0.01, ***p < 0.001.

^2^Negative change scores indicate improvement.

### Estimates of Minimum Important Differences

There is no one best way to estimate the MID for a PRO measure [21,34]. Table 8 contains three different anchor-based methods for estimating the MID based on data from this study. MID-1 contains the estimate obtained from the scores from the "near-responders," shown as the PASI 25-PASI 49 group in Table 7; MID-2 contains the estimate corresponding to "partial responders" in the same table 7. MID-3 corresponds to the difference between non-responders for the PGA (defined as patients who had no change in score or a decrease in score by one point on this 7-point scale) and minimal responders for this same measure (defined as patients who improved by 1 or 2 points). The distribution-based estimates, the SEM and one-half the standard deviation of baseline scores are also reported in Table 8.

**Table 8:** Estimates of MCID for PRO measures

**Change in Measure**	**MID-1: PASI Improvement 25–49%**	**MID-2: PASI Improvement 50–74%**	**MID-3: Difference Between Non-Responders (n = 34) and Minimal Responders (n = 41) on PGA**	**SEM**	**0.5 SD**
**DLQI Total Score**	-4.05 (4.95)	-6.95 (5.71)	-5.69	2.33	3.59
**SF-36**					
Physical Functioning^1^	N/A	N/A	N/A	N/A	N/A
Role-Physical	3.41 (51.35)	14.29 (31.20)	10.51	15.02	18.78
Bodily Pain	11.59 (22.96)	16.76 (22.74)	9.05	10.76	12.69
General Health	4.41 (18.37)	9.19 (11.29)	4.97	9.67	10.31
Vitality	4.77 (20.44)	6.90 (17.43)	6.54	8.22	11.40
Social Functioning	-1.70 (27.36)	13.10 (17.44)	13.62	10.67	13.78
Role-Emotional	-7.58 (38.40)	17.46 (34.35)	24.71	14.59	17.70
Mental Health	0.18 (18.66)	9.52 (13.53)	4.90	6.10	9.65
Physical Summary Score (PCS)	3.91 (10.88)	2.57 (7.78)	0.51	2.71	5.12
Mental Summary Score (MCS)	-1.82 (11.38)	6.05 (6.90)	6.61	3.89	5.61
**EQ-5D**					
Index Score	0.10 (0.24)	0.20 (0.21)	0.09	0.22	0.14
VAS-General Health	3.82 (22.07)	8.43 (11.24)	4.59	N/A	10.34

Note: MID-1 corresponds to the score for the PASI 25–49 group; MID-2 corresponds to the score for the PASI50-74; for MID-3 and MID-4, reliability estimates for computing SEM were obtained from the data in this study for the DLQI and from estimates found in the literature for the SF-36 and EQ-5D.

^1^MID estimates are not provided for the SF-36 Physical Function domain since there were not significant differences among responder groups.

Estimates for the DLQI MID ranged from 4.05 (for MID-1) to 6.95 (for MID-2), while the SEM was 2.33 and one-half standard deviation was 3.59. The MID results for the SF-36 PCS ranged from 0.51 (for MID-3) to 3.91 (for MID-1), with the SEM estimated as 2.71 and one-half standard deviation estimates as 5.12. For the MCS, the MID estimates included a decrease of 1.82 points based on a PASI improvement of 25–49%, but the other two MIDs were 6.05 and 6.61, respectively. The SEM for the MCS was 3.89 and one-half standard deviation was 5.61. Consistent with the MCS findings, decreases were observed for the Role-Emotional and Social Functioning domains for the MID-1 definition. The differences between non-responders and minimal responders ranged from 4.90 for Mental Health to 24.71 for Social Functioning (Table 8). The results for the EQ-5D index score demonstrated an MID ranging from 0.09 (for MID-3) to 0.20 (for MID-2). For the EQ-5D VAS, the available estimates ranged from 3.82 (MID-1) to 8.43 (MID-3).

## Discussion

A Phase II randomized clinical trial of adalimumab in moderate to severe plaque psoriasis provided the opportunity to evaluate the validity and responsiveness to clinical change of three PRO assessment instruments – one dermatology-specific instrument and two general health status instruments – all used as endpoints in the study. All analyses were performed on a blinded basis, since the main focus of these secondary analyses was on the psychometric qualities of the PRO instruments.

Although developed for a general population with dermatologic diseases, the DLQI has most frequently been applied to patients with plaque psoriasis [27]. More recently, the DLQI has been used as an endpoint in clinical trials involving the newer class of biologics for treatment of moderate to severe psoriasis, including alefacept [6,7], etanercept [9,10], and efalizumab [8,11]. The present study further establishes the reliability and validity of the DLQI and its responsiveness to change in the clinical status of patients over the course of a 12-week clinical trial, confirming previous findings [28]. Changes in the DLQI total score demonstrated significant and sizeable correlations with independently obtained physician-assessed changes in the clinical statuses of patients. This indicates that the alleviation of psoriatic signs, as determined by clinical assessments, results in significant and marked improvement in dermatologic-related functional limitations and quality of life in patients with moderate to severe plaque psoriasis. Based on this study, the DLQI is a psychometrically sound and responsive measure of psoriasis-specific outcomes that captures more comprehensively the impact of clinical signs and symptoms on patient well-being.

Data were also used to derive estimates of the MID of the DLQI. Although the MID is defined as the smallest difference that a patient would perceive as beneficial, there were no patient-based assessments of change in this study. Hence, lacking a patient-based anchor, the data do not provide the basis for determining the smallest score that a patient would perceive as beneficial. We used both the PASI and the PGA, as well as two distributional approaches to derive estimates of the MID of the DLQI. These estimates ranged from 2.33–6.95. However, we believe that the PASI 50 is too conservative for estimating the minimum change that patients will find beneficial. Therefore, we believe the estimate based on PASI improvement of 25–49% or between non-responders and minimal responders provide better estimates of MID. Therefore, the results indicate that the MID is in the range of approximately 2.3–5.7, which is slightly higher than the range of estimates derived from Shikiar et al. [28] in an analysis of two clinical trials involving another psoriasis therapy. The distributional approaches resulted in the lowest estimates of MID for the DLQI, but it should be noted that the distributional approach to estimating the MID is considered supportive of the anchor-based methods [22,35]. For example, the one-half standard deviation estimate is certainly clinically meaningful, but is likely not a minimum magnitude of change. Finally, the range of estimates incorporates another previous estimate of the MID of the DLQI of 5.0 [36].

Two general PRO measures were used in this study. In general, the EQ-5D index and VAS scores demonstrated higher correlations than the SF-36 scale scores with the clinical endpoints (Table 5). However, the responsiveness of these two EQ-5D scores was generally the same as the responsiveness of most of the SF-36 scores. Nonetheless, this study demonstrated that the EQ-5D performs at least as well as the SF-36 as a non-dermatologic specific PRO measure for this sample of moderate to severe psoriatic patients.

Although most of the SF-36 scores showed improvements associated with clinical outcomes, the MCS, Social Functioning, and Role-Emotional domain scores demonstrated decreases in the PASI 25–49% group. These findings may have been driven by several outliers and the relatively small sample size for this group. Alternatively, given that Bodily Pain and other physical domains may be more related to the signs and symptoms of psoriasis than Role-Limitations and Social Functioning, small improvements in PASI scores may not be directly associated with changes in these PRO domains. That is, larger changes in clinical outcomes may be needed to significantly impact the areas of physical function and well-being. This idea seems to be supported by the observed changes in the PASI 50–74% and other analyses. However, the SF-36 domain and summary scores demonstrated consistently reasonable validity and were correlated with clinical endpoints and DLQI scores.

The SF-36 PCS and MCS scores demonstrated good evidence of validity and responsiveness in this sample of patients with moderate to severe plaque psoriasis. There were demonstrable associations between changes in PASI score categories and changes in PCS scores, with the largest improvements seen in the PASI75 responder groups. The MID estimates for the PCS were in the range of 0.51–3.91, with the best estimate at approximately 2.5 points. The SEM estimate (2.71) also supports this range of MID values for the PCS. These results are consistent with previous research on the PCS scores in rheumatoid arthritis and other chronic diseases [29, 37]. The MID findings for the MCS were somewhat weaker, but there is evidence that a change of 4–6 points is certainly clinically meaningful. The MID for the EQ-5D index score was in the range of 0.09–0.22.

Given the impact of psoriasis on the functional ability of patients the importance attached to assessing physical function in psoriasis patients, the results of the present study provide positive support for the use of a dermatology-specific health-related PRO measure, the DLQI, in the assessment of psoriasis and responses to treatment. In addition, the correlation of SF-36 and DLQI indicates that disease-related changes in the SF-36 are largely dependent on two specific domains, Bodily Pain and Social Functioning. It appears that the DLQI total score, as a single index score, adequately captures the functional and psychosocial impact of moderate to severe plaque psoriasis. Further, the DLQI does so in a way that is substantially more responsive than the general health-related quality of life measures used to assess changes in patients' underlying clinical statuses. The importance of the DLQI in measuring psoriasis patients' disease statuses, both at baseline and after treatment, is underlined by recent UK guidelines that recommend the DLQI serve both as an indicator of biologic therapy need and adequate treatment response [14].

There were several limitations to the present analysis. The first relates to sample size and selection. The sample was limited to those meeting the inclusion/exclusion criteria if this Phase II clinical trial. Since this was a Phase II study, the sample size was smaller (N = 147) than typical Phase III studies involving moderate to severe psoriasis, thereby requiring even one to use even greater caution in extrapolating the results of this analysis. Other applications of the PRO instruments (e.g., other clinical settings or settings including non-biologic treatments) might not involve the same exclusions. Therefore, generalizability of these results may not be applicable to all clinical settings. Second, the DLQI is not the only dermatology-specific instrument to assess the impact of psoriasis on physical function and psychosocial factors. Other instruments have been developed [38, 39], but have not been used as frequently as the DLQI in psoriasis trials. Nonetheless, results reported here do not indicate whether the DLQI has relative advantages or disadvantages to these instruments. Finally, given that the MID denotes the minimum change that a patient would find beneficial, anchoring the estimates of MID to patient assessments of severity or change would prove useful, and the current Phase II trial did not include such assessments.

## Conclusion

The findings of this study highlight the importance of capturing PRO measures in clinical trials of moderate to severe plaque psoriasis. This analysis provides additional evidence supporting the psychometric qualities and responsiveness of the DLQI as a disease-specific measure of PROs in psoriasis. The DLQI MID was determined as ranging from 2.3–5.7 points. While the DLQI provides the most reliable measure of clinical change, the data from this study demonstrate that the SF-36 and EQ-5D performed well as general measures of health status outcomes. While the SF-36 has been used in previous studies comparing psoriasis treatments [6,7,30], to date, there have been few applications of the EQ-5D in clinical trials of patients with moderate to severe plaque psoriasis. The results of this study indicate that these two instruments should be considered as a general health outcome measure in future clinical trials.

## Competing interests

The work reported here was performed under contract by the United BioSource Corporation to Abbott Laboratories. Authors 1, 4, and 5 are employees of the United BioSource Corporation, which performed this work under contract. Authors 2 and 3 are current employees of Abbott Laboratories.

## Authors' contributions

RS was responsible for the planning and analysis of the research reported here. MKW provided key analysis and interpretation of the data, and revised the manuscript critically for important intellectual content. MMO contributed important clinical insights into the research. CST contributed suggestions for analysis and provided analytical support for the research. DAR contributed to the analysis and interpretation of the research and reviews of the manuscript. All authors reviewed and approved this manuscript prior to submission.
